# A virtual reality extended neuropsychological assessment for topographical disorientation: a feasibility study

**DOI:** 10.1186/1743-0003-4-26

**Published:** 2007-07-11

**Authors:** Francesca Morganti, Andrea Gaggioli, Lorenzo Strambi, Maria Luisa Rusconi, Giuseppe Riva

**Affiliations:** 1 Applied Technology for Neuro-Psychology Lab, Istituto Auxologico Italiano, Via G. Pelizza da Volpedo 41, I-20149 Milano, Italy; 2 Institute of Psychology and Sociology of Communication, University of Lugano, Via G. Buffi 13, CH 6904 Lugano, Switzerland; 3 Department of Human Science, University of Bergamo, Piazzale S. Agostino 2, I-24129 Bergamo, Italy

## Abstract

**Background:**

Topographical disorientation represents one of the main consequences of brain injury. Up to now several methodological approaches have been used in the assessment of the brain injured patient's navigational abilities showing a moderate correlation with the impairments observed in everyday contexts.

**Methods:**

We propose a combination of standardized neuropsychological tests and a more situated virtual reality-based assessment for the evaluation of spatial orientation in brain injured patients.

**Results:**

When tested with this virtual reality integrated procedure patients showed performance and execution times congruent with their neuropsychological evaluation. When compared to a control group, patients revealed significantly slower times and greater errors in solving virtual reality based spatial tasks.

**Conclusion:**

The use of virtual reality, when combined with classical neuropsychological tests, can provide an effective tool for the study of topographical disorientation.

## Background

Topographical disorientation, when associated with a complex cognitive impairment, represents one of the main consequences of extended brain injury and occurs most frequently from damage in the parietal/temporal/occipital regions [1-5].

Patients with topographical disorientation revealed impairment in the capacity to orient oneself in and to cope with everyday environments. Despite this statement, up to now most of the evaluation tools for topographical disorientation introduced laboratory methodologies in order to understand how people explore three-dimensional spaces [6-12].

Laboratory evaluation does not support an immediate and direct interaction generally possible in the daily environment, and constitutes an important bias for the evaluation of spatial impairments in brain damage, often making clinicians desire a more ecological approach. Ecological methods, in fact, might help clinicians to observe the strategy used by a patient to explore a natural environment, and to understand the characteristics of the environment utilized to help in familiarization. On the other hand, the observation of a patient in daily contexts generates many questions about the reliability and interpretation of collected data. In order to avoid biases the design of an effective assessment tool that links a reliable evaluation methodology with a more situated observation of spatial behaviors appears to be still needed.

A promising way could be to integrate classical evaluation tools with computer-based interactive ones, such as virtual reality, in order to evaluate the navigational capacity of complex environments by neurological patients by observing the types of spatial representations that a patient is able to produce in order to adaptively interact with space within a given activity.

Virtual reality (VR) environments, in fact, constitute an interesting opportunity for the evaluation of topographical disorientation, providing a representation of a dynamic nature and interactive environments [13,14]. In particular, we propose that from patients' active interaction with virtual reality simulations it is possible to clearly understand how the ability to organize spatial knowledge into a cognitive map is preserved.

The study of spatial cognition has provided evidence of how it can be represented in a cognitive map which can be of the "route" or "survey" type [15-18]. The "route" map is made up of representations of an egocentric nature. "Survey" maps, on the other hand, are representations of a higher level, organized according to an allocentric perspective. Studies in clinical neuropsychology showed how the processing of topographical information may be conducted within an egocentric or allocentric coordinate frame that are mainly comparable to route and survey knowledge organization [19,20].

According to a particularly theoretical orientation, the capability of learning spatial relationships in a large scale environment, and of organizing them into "route" or "survey" types, is influenced not so much by a major familiarity with the environment, but by a series of characteristics of the specific environment, capable of assuming a functional role with the activities an agent performs or is going to perform inside it [21-23]. We propose an integrated evaluation method in which neuropsychological spatial ability evaluation will be extended with more situated computer based tools that allow the assessment of spatial orientation during the interaction with complex 3D environments.

## Methods

### Materials

In order to evaluate patients' spatial abilities with "paper and pencil" tests a neuropsychological assessment was performed. For general cognitive level identification Progressive Raven's Matrixes (PM47) were employed. Token test, Boston Naming test and phonemic and semantic Fluencies were used for verbal abilities evaluation. Memory ability was assessed through Digit and Corsi's span where attention and executive functions were evaluated through the Trial Making test, Attentive matrixes and the Tower of London test. Finally, spatial ability evaluation required the Rey's complex figure, Poppelreuter's test, Benton's line orientation test, Street's Completion test and the Elithorn's Perceptual Maze test.

We extended this evaluation by addition of the VR-Maze test and VR-Road Map. We developed these tools [24] to support the assessment of spatial orientation during the interaction with daily complex environments.

**Figure 1:**
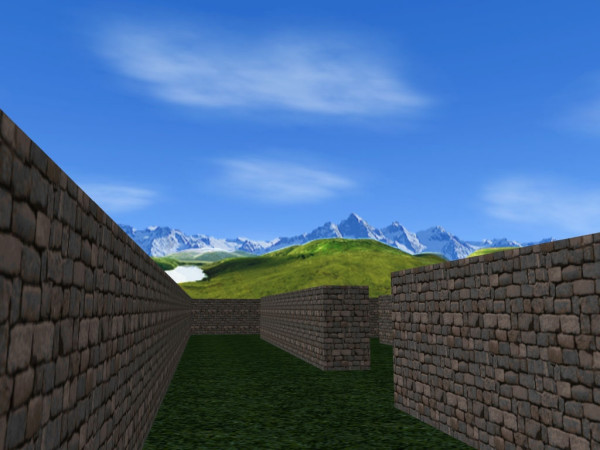
The VR Maze test.

The VR-Maze test, depicted in figure 1, is based on the Wisc-R Maze subtest [25]. Patients were requested to firstly perform the allocentric paper and pencil version of eight mazes, to memorize it and after to find the right way into the equivalent egocentric VR version of the maze. Performance times are recorded for each of the eight mazes.

**Figure 2:**
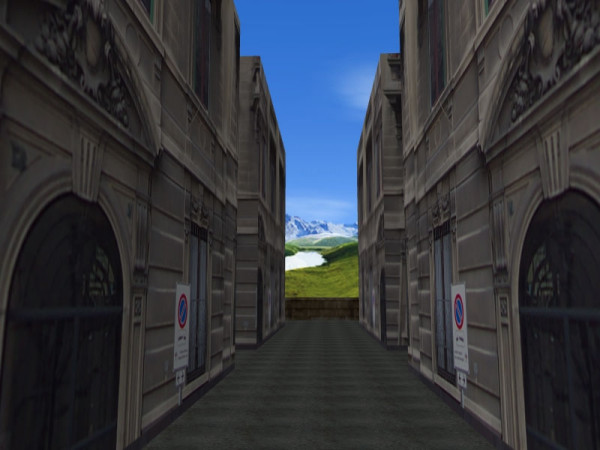
The VR Road Map test.

The VR-Road Map test, depicted in figure 2, is a virtual reality version of the Road Map Test [26], in which the paper and pencil version is turned into a simulated and actively explorable city. No landmark objects were provided as navigation aids and all the buildings have the same texture. Patients are requested to follow the designated route into the virtual environment using the survey paper and pencil version of the test as a guide map. Performance time and errors were recorded.

**Figure 3:**
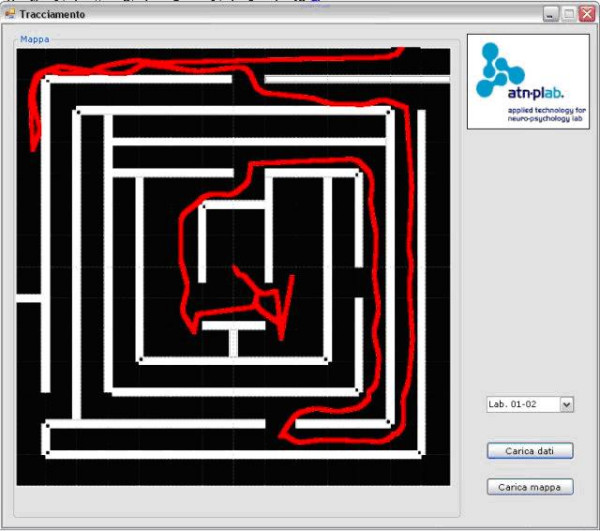
The VR Maze Test tracking tool.

**Figure 4:**
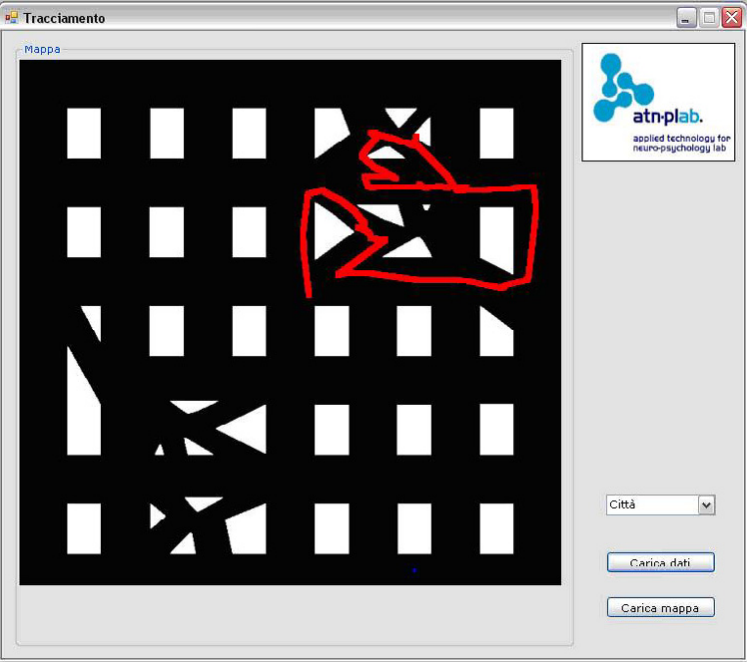
The VR Road Map test tracking tool.

For both the VR systems the possibility to track user's spatial behaviour is provided. Snapshots of the tracking tool is provided in figure 3 and 4.

### Participants

Four male patients with brain damage (mean age = 31.7 years, sd = 1.5 and mean years of school attendance = 16.7 years, sd = 2.5) were tested using paper and pencil neuropsychological evaluation and with the VR-integrated procedure. Patients' performance on VR-Maze test and VR-Road Map test were compared to a control group (10 male participants, with a mean age of 29.3 years, sd = 4.5 and 15.5 years of school attendance, sd = 2.6).

## Results

**Figure 5:**
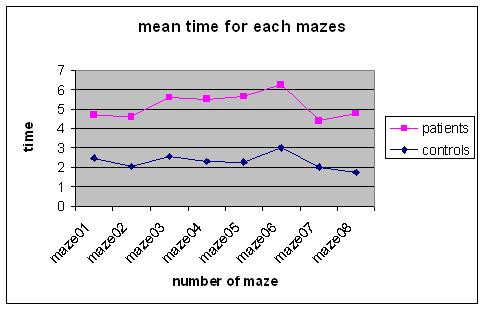
VR Maze execution times.

**Figure 6:**
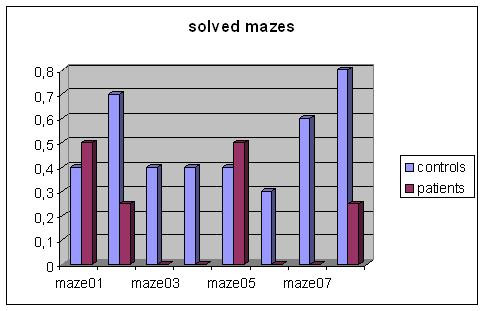
Number of solved VR Mazes.

All the patients appeared to have deficiencies in spatial knowledge organization based on their neuropsychological assessment. They were all at the same level of deficits in the organization of spatial knowledge. With regard to the VR Maze test, in the comparison to the control group, patients revealed significantly slower execution times in solving tasks (T-Test = .677; p = .013) and were able to solve four out of the eight mazes as depicted in figure 5 and 6.

**Figure 7:**
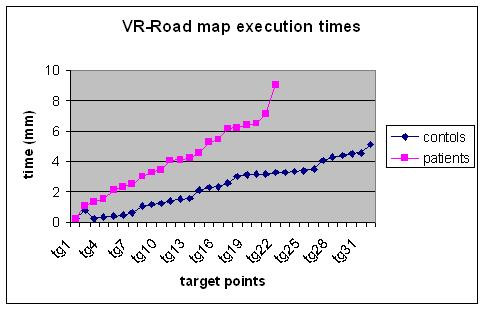
VR Road Map execution Times.

Figure 7 shows how patients' execution times in the VR-Road Map tests appeared to be higher than those of the control group and all patients were unable to reach the final target point. The control group did not show any difficulty in doing this.

## Discussion and Conclusion

Both in the VR-maze and in VR-Road Map tests, patients' execution times were higher than those of the control group. Moreover, patients' performance in the VR-maze and in the VR-Road Map tests were lower when compared to the control group.

These results underline how patients' performance and execution times in VR tests are congruent with their neuropsychological evaluation.

VR environments provide the possibility of an egocentric interaction that will highlight patient's difficulty in the translation of survey spatial knowledge into a route one. It could be considered the main feature of topographical disorientation disease.

The integration of virtual reality with traditional evaluation methods may provide an interesting alternative to paper and pencil-based approaches, thereby contributing to an improvement in the evaluation of topographical disorientation [27].

The observation of patients' performance allows an assessment of their difficulty in the translation of survey knowledge to route knowledge. In accordance with their impairment, they were able to memorize sequences of paths when provided in a top-view perspective (the survey one). This residual ability is usually assessed using classical neuropsychological evaluation since paper and pencil materials provide spatial relations mainly from a top-down view. In contrast, the integrated approach we have proposed allows an analysis of the survey-route translation of knowledge. In conclusion, the use of virtual reality appears to provide an effective and objective tool for the study of topographical disorientation when combined with classical neuropsychological tests.

## Competing interests

The author(s) declare that they have no competing interests.

## Authors' contributions

FM, AG, LS, MLR and GR equally participated to the design and execution of the study. All authors read and approved the final manuscript.
